# Comparison of two preprocessing methods for ^18^F-Flortaucipir PET quantification in Alzheimer’s disease

**DOI:** 10.1007/s00259-025-07452-3

**Published:** 2025-07-19

**Authors:** Elif Harput, Debora Elisa Peretti, Max Scheffler, Nicholas J. Ashton, Kaj Blennow, Henrik Zetterberg, Ruben Smith, Giovanni B. Frisoni, Valentina Garibotto, Cecilia Boccalini

**Affiliations:** 1 Laboratory of Neuroimaging and Innovative Molecular Tracers (NIMTlab), Geneva University Neurocentre and Faculty of Medicine, University of Geneva, 1205 Geneva, Switzerland; 2 Division of Radiology, Geneva University Hospitals, Geneva, Switzerland; 3 Department of Psychiatry and Neurochemistry, Institute of Neuroscience and Physiology, the Sahlgrenska Academy at the University of Gothenburg, Mölndal, Sweden; 4 Banner Alzheimer’s Institute and University of Arizona, Phoenix, AZ USA; 5 Banner Sun Health Research Institute, Sun City, AZ 85351 USA; 6 Centre for Age‑Related Medicine, Stavanger University Hospital, Stavanger, Norway; 7 Clinical Neurochemistry Laboratory, Sahlgrenska University Hospital, Mölndal, Sweden; 8 Paris Brain Institute, ICM, Pitié‑Salpêtrière Hospital, Sorbonne University, Paris, France; 9 Neurodegenerative Disorder Research Center, Division of Life Sciences and Medicine, and Department of Neurology, Institute On Aging and Brain Disorders, University of Science and Technology of China and First Affiliated Hospital of USTC, Hefei, People’s Republic of China; 10 Department of Neurodegenerative Disease, UCL Institute of Neurology, Queen Square, London, UK; 11 UK Dementia Research Institute at UCL, London, UK; 12 Hong Kong Center for Neurodegenerative Diseases, Clear Water Bay, Hong Kong, China; 13 Wisconsin Alzheimer’s Disease Research Center, University of Wisconsin School of Medicine and Public Health, University of Wisconsin-Madison, Madison, WI USA; 14 Clinical Memory Research Unit, Lund University, Lund, Sweden; 15 Memory Clinic, Skåne University Hospital, Malmö, Sweden; 16 Geneva Memory Centre, Department of Rehabilitation and Geriatrics, Geneva University Hospitals, 1205 Geneva, Switzerland; 17 Laboratory of Neuroimaging of Aging (LANVIE), University of Geneva, 1205 Geneva, Switzerland; 18 Division of Nuclear Medicine and Molecular Imaging, Geneva University Hospitals, 1205 Geneva, Switzerland; 19 CIBM, Centre for Biomedical Imaging, University of Geneva, 1205 Geneva, Switzerland

**Keywords:** Tau-PET, Semi-quantification, Reference space, Alzheimer’s disease

## Abstract

**Background:**

Tau-Positron Emission Tomography (PET) has become central in Alzheimer’s disease (AD) research and clinical settings. Multiple preprocessing pipelines for tau-PET quantification have been described, with satisfactory performance but direct comparisons remain scarse. Our study evaluates the comparability of two commonly used PET preprocessing methods, respectively in native and standard spaces, in quantifying tau deposition and in their ability to discriminate AD patients.

**Methods:**

209 subjects were included from the Geneva memory clinic including cognitively unimpaired (CU) individuals, mild cognitive impairment (MCI) and dementia patients. Images were processed in native and standard space using inferior cerebellar grey matter as reference region. Standardized uptake value ratios (SUVR) were extracted from AD-specific regions. Correlations between SUVR obtained by different methods and plasma biomarkers were assessed. ROC analyses compared the ability of the two methods to discriminate visually assessed tau status, amyloid-positive cognitively impaired from amyloid-negative CU, and subjects with declining cognition over time.

**Results:**

SUVR from the two methods were strongly correlated across all regions. However, SUVR values obtained with standard space method showed higher values. SUVR in the medial temporal lobe from native space processing provided a greater accuracy in discriminating positive scans and identifying subjects with cognitive decline. For all other analyses methods performed equally well. The correlation with plasma biomarkers was comparably high with both methods.

**Conclusion:**

While preprocessing in native and standard space is adequate for quantifying ^18^F-Flortaucipir PET and for discriminating AD patients, higher accuracy can be obtained in the mesial temporal regions and to predict cognitive decline using processing in native space.

**Supplementary Information:**

The online version contains supplementary material available at 10.1007/s00259-025-07452-3.

## Introduction

Alzheimer’s disease (AD) is characterized by the accumulation of amyloid-beta (Aβ) plaques and tau neurofibrillary tangles, accompanied by a progressive neurodegenerative process leading to neuronal and synaptic loss [1]. Positron emission tomography (PET) serves a pivotal tool for the in vivo visualisation and quantification of AD pathology [2], with tau-PET measuring tau pathological changes [3] that have been strongly associated with cognitive impairment and decline in AD. Tau-PET outperformed other AD biomarkers in predicting cognitive worsening [4–6], overall supporting its relevance as a biomarker for disease staging and prognosis [4, 7, 8].

Despite the emerging relevance of tau-PET imaging, no consensus exists on a reference pipeline for the quantification of tau-PET. Variability in PET quantification arises from multiple methodological factors, including different tracers, choice of target/reference regions, native vs standard space processing or selection of different brain atlases [9, 10]. The reference region selection depends on the radiotracer used [11]. For most tau tracers, cerebellar grey matter is chosen [12], using frequently the inferior cerebellar grey matter or the cerebellar crus.

The reference space selection has been demonstrated to have impact on quantification with amyloid-PET where native space processing can improve the accuracy of standardized uptake value ratios (SUVR) [13], but this has never been explored for tau-PET using ^18^F-flortaucipir neither with any other tracer. In standard space processing tau-PET images were spatially normalized to a common template, in many cases the Montreal Neurological Institute (MNI) template, whereas, in native space processing images are analysed in the individual subject’s anatomical space. Standard space processing facilitates voxel-wise analysis, whereas native space preserves anatomical information, and it is recommended in volumetric measurements [14]. Since native space method may require more computational resources and longer preprocessing times [13, 14], standard space method could be a viable alternative when MRI is unavailable or to reduce the time required for processing [15].

Given the lack of evidence regarding the impact of reference space selection on tau-PET quantification, the present study aims to compare two different commonly used preprocessing methods for ^18^F-flortaucipir PET, respectively in native and standard reference space. The pipeline in the native space processing is commonly used in research [16, 17], while most of clinical research series are processed in standard space [4, 18, 19]. Our aim was to examine whether there are differences between the results obtained by processing in native space and standard space across different target regions, in terms of the quantitative parameters obtained and their association with clinical and biological parameters, in a memory clinic population. As reference standards we used the visual assessment of tau-PET images, phospho-tau species measured in plasma, the presence of amyloid pathology and clinical severity, as well as cognitive decline.

## Methods

### Participants

This study included 209 subjects from the Geneva memory clinic, including cognitively unimpaired subjects (CU, *N* = 62), cognitively impaired patients (CI) with mild cognitive impairment (MCI, *N* = 113) and dementia (*N* = 34). Each participant underwent clinical and neurological assessment, neuropsychological testing, and MRI scan. Inclusion criteria was to have undergone at least a tau-PET scan and a Mini-Mental State Examination (MMSE) within one-year timeframe. A subset of participants (*n* = 182) also underwent amyloid-PET within one year of the tau-PET assessment, and Aβ status was defined by visual rating by nuclear medicine experts. Follow-up MMSE was available for a subgroup of 126 participants at 23 ± 12 months. We classified subjects into decliners or stable based on the change from MMSE at baseline and MMSE at the last follow-up considering follow-up duration and participant’s age [20].

The study procedures were approved by the local ethics committee and conducted in accordance with the principles of the Declaration of Helsinki and the International Conference on Harmonization Good Clinical Practice. All participants provided informed consent before taking part in the study.

## Imaging acquisition and preprocessing

### MRI and PET acquisition

T1-MRI images were performed at Division of Radiology of Geneva University Hospitals and all details are reported in Supplementary Materials (S1).

PET scans were acquired at the Division of Nuclear medicine and Molecular Imaging at Geneva University Hospitals. Tau-PET imaging was acquired using ^18^F-flortaucipir tracer, while amyloid-PET images were acquired using either ^18^F-florbetapir (FBP) or ^18^F-flutametamol (FMM) tracers. All details of PET acquisition are reported in Supplementary Materials (S1).

### MRI and Tau-PET processing

All tau images have been processed by two different processing methods, involving two different spaces, with the aim of comparing the two different outputs.

***Native space processing:*** T1-weighted MRI images were segmented using FreeSurfer. Each participant’s mean tau-PET image was coregistered to their corresponding native T1-weighted MRI images. Images were intensity-normalized using the inferior cerebellar grey matter as a reference region, yielding in SUVR images. FreeSurfer parcellations were applied to extract mean SUVR values from all regions in the Desikan-Killiany atlas [21] in native space using PETSurfer.

***Standard space processing***: PET standard space processing was performed using Statistical Parametric Mapping (SPM,12, Wellcome Trust centre for Neuroimaging, London, UK), running in MATLAB R2018b version 9.5 (MathWorks Inc., Sherborn, MA, USA). MRI 3D T1 images were aligned to a reference plane passing through the anterior and posterior commissures, segmented into different compartments, and normalized to the MNI space using tissue probability maps. PET images were aligned to the subject’s respective T1 MRI scan and normalized to MNI space using the transformation matrix that was generated during the registration of the MRI images to the standard space. Standardized intensity normalization was performed using inferior cerebellar grey matter as a reference region resulting in SUVR images. As the cerebellar crus has also been largely used as reference region for standard space processing, we also include data derived using it as supplementary analysis. SUVR were extracted using Desikan-Killiany atlas.

#### AD-related target regions

After obtaining the SUVR for the Desikan-Killiany atlas regions with both processing methods, global tau SUVR were calculated from specific AD regions of interest (ROIs): (a) medial temporal lobe (MTL), (b) early to late AD related areas (global AD meta-ROI) according to [22], (c) early AD meta-ROI as the most discriminative for tau status for cognitively normal population according to [23], (d) lateral temporal ROI, (e) and Braak regions. SUVR in meta-ROIs and Braak regions were calculated using a weighted approach, mean among all included regions, accounting for the volume (native space processing) or the size (standard space processing) of each region. All regions included in AD ROIs and Braak regions are reported in Supplementary Materials (S2).

### Plasma biomarkers

Plasma biomarkers (Plasma Aβ42 and Aβ40: *N* = 128; Plasma p-tau181 and p-tau23: *N* = 121; Plasma p-tau217, *N* = 47) were available for subgroups of patients. All details about plasma collection are reported in Supplementary Materials (S3).

### Statistical Analysis

Demographic differences across diagnostic groups were assessed using Kruskal–Wallis tests. For each meta-ROI and Braak region, Pearson correlation coefficients were calculated between the SUVR obtained by the two preprocessing methods across the entire sample. To compare the mean SUVR values obtained from native and standard space methods, paired t-tests were conducted, with significance set at 0.05. To quantify the amount of tau-PET positive subjects for both methods, first, threshold values for tau positivity were calculated with Youden’s test for SUVR in native and standard space methods using as reference the visual classification of tau pathology as assessed by a nuclear medicine expert (VG). Receiver operating characteristic (ROC) analyses were performed to compare SUVR obtained using the two methods in discriminating visual tau status, A+ CI/A-CU status, and declining status. The DeLong test was used to compare areas under the curve (AUCs). Pearson correlations were computed for testing the correlations between plasma biomarkers and SUVR in early and global AD meta-ROIs obtained with the two methods and differences between standard space and native space correlations were compared using “cocor” package in R [24].

Similar analyses have been performed in parallel to compare SUVR obtained with standard space processing method with two different commonly used reference regions (inferior cerebellar grey matter and cerebellar crus).

Given the different spatial resolution of PET scanners, sensitivity analyses were run within two subgroups acquired with PET scanners with different resolution (Biograph mCT, *N* = 139, and Biograph Vision 600 Edge, *N* = 66). ROC analyses were conducted to assess the ability of SUVR obtained using the two methods in discriminating visual tau status, A + CI/A-CU status, and cognitive decline status within the two groups. To ensure comparability in sample size, 66 subjects were randomly selected from a total of 139 individuals scanned with Biograph mCT, of which 39 individuals had available follow-up MMSE scores, to match the number of follow-up cases in the group scanned with the Biograph Vision.

All statistical analyses were performed with R version 4.0.2 (R Foundation for statistical computing, https://www.r-project.org/).

## Results

### Participants

Demographic and clinical variables are presented in Table 1. Out of 209 participants, the majority were MCI patients (54%). The mean age was 70.34 ± 9.1 and MMSE 25.52 ± 4.4. Age and MMSE differed significantly between disease stages (Table 1). The rate of amyloid and tau positivity frequency, and all plasma levels except for plasma p-tau231 (*p* = 0.07) were significantly higher in the dementia group compared to the other diagnostic groups (*p* < 0.05).

**Table 1:** Demographic characteristics of participants

	CU (*n* = 62)	MCI (*n* = 113)	DEM (*n* = 34)	Whole sample (*n* = 209)	*p*-value
Age (years)	67.67 ± 10.9	72.13 ± 7.5	69.26 ± 9.6	70.34 ± 9.1	0.03
Sex (F/M)	35/27	52/61	20/14	107/102	0.26
Education (years)	14.47 ± 4.6	13.74 ± 3.9	12.21 ± 3.9	13.71 ± 4.2	0.07
MMSE	27.75 ± 2.2	26.26 ± 2.4	20.08 ± 6.5	25.52 ± 4.4	< 0.001^a^
Imaging Biomarkers					
Amyloid Status (visual read) ( ±)	10/46	67/36	27/5	104/87	< 0.001^b^
Tau Status (visual read) ( ±)	7/55	51/60	25/8	83/123	< 0.001^b^
AT Status (A-T-/A + T-/A + T + /A-T +)	46/5/5/0	32/23/43/3	5/3/24/0	83/31/72/3	< 0.001^b^
Centiloid	10.46 ± 30.7	52.32 ± 47.8	73.80 ± 44.3	43.55 ± 48.3	< 0.001^b^
Plasma Biomarkers					
Plasma Aβ 42/40	0.07 ± 0.02	0.06 ± 0.01	0.05 ± 0.01	0.06 ± 0.01	0.0003^a^
Plasma p-tau181	16.01 ± 8.3	21.67 ± 11.1	24.34 ± 9.4	20.13 ± 10.5	0.003^b^
Plasma p-tau231	10.49 ± 4.9	12.42 ± 5.9	14.04 ± 7.9	11.97 ± 5.9	0.07
Plasma p-tau217	13.22 ± 7.8	7.14 ± 4.2	13.22 ± 7.8	6.8 ± 5.4	0.001^b^

*CU* cognitively unimpaired, *MCI* mild cognitive impairment, *DEM* dementia, *MMSE* Mini-Mental State Examination, *Aβ* Amyloid Beta

Table indicates mean ± standard deviation for continuous variables and number of subjects for dichotomous variables

^a^ Dementia < MCI < CU; ^b^ Dementia > MCI > CU

### Comparison of different processing methods

**Fig. 1:**
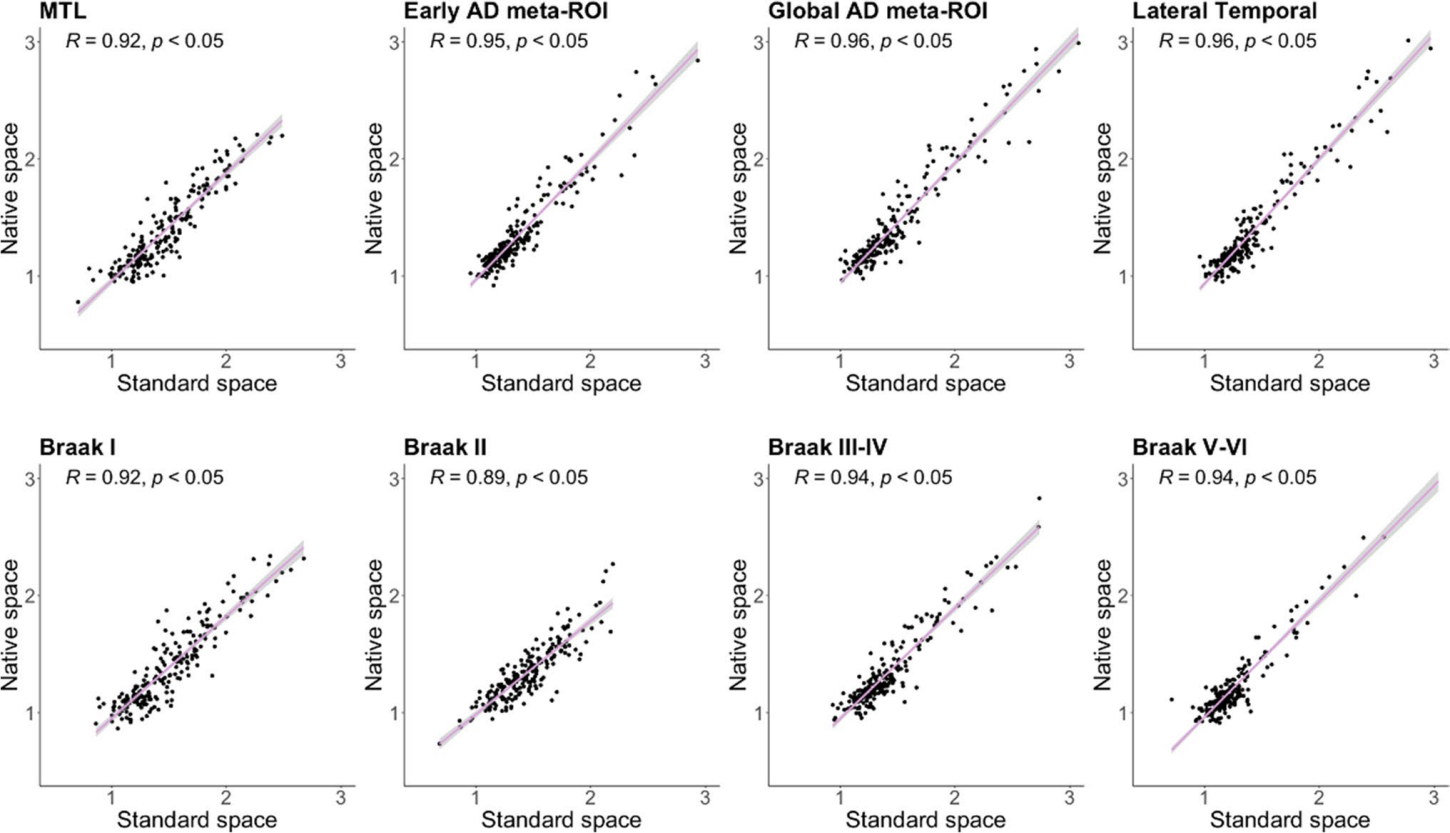
Correlation between tau SUVR obtained by native and standard space methods. Scatter plots of native and standard space SUVR across the meta-ROIs and Braak regions for the whole sample. Abbreviations: *MTL* medial temporal lobe, *AD* Alzheimer’s disease, *ROI* region of interest, *SUVR* standardized uptake value ratio

SUVR obtained by the two processing methods in all analysed meta-ROIs and Braak regions showed strong and significant correlations (r > 0.9, *p* < 0.05) in the whole sample (Fig. 1).

**Fig. 2:**
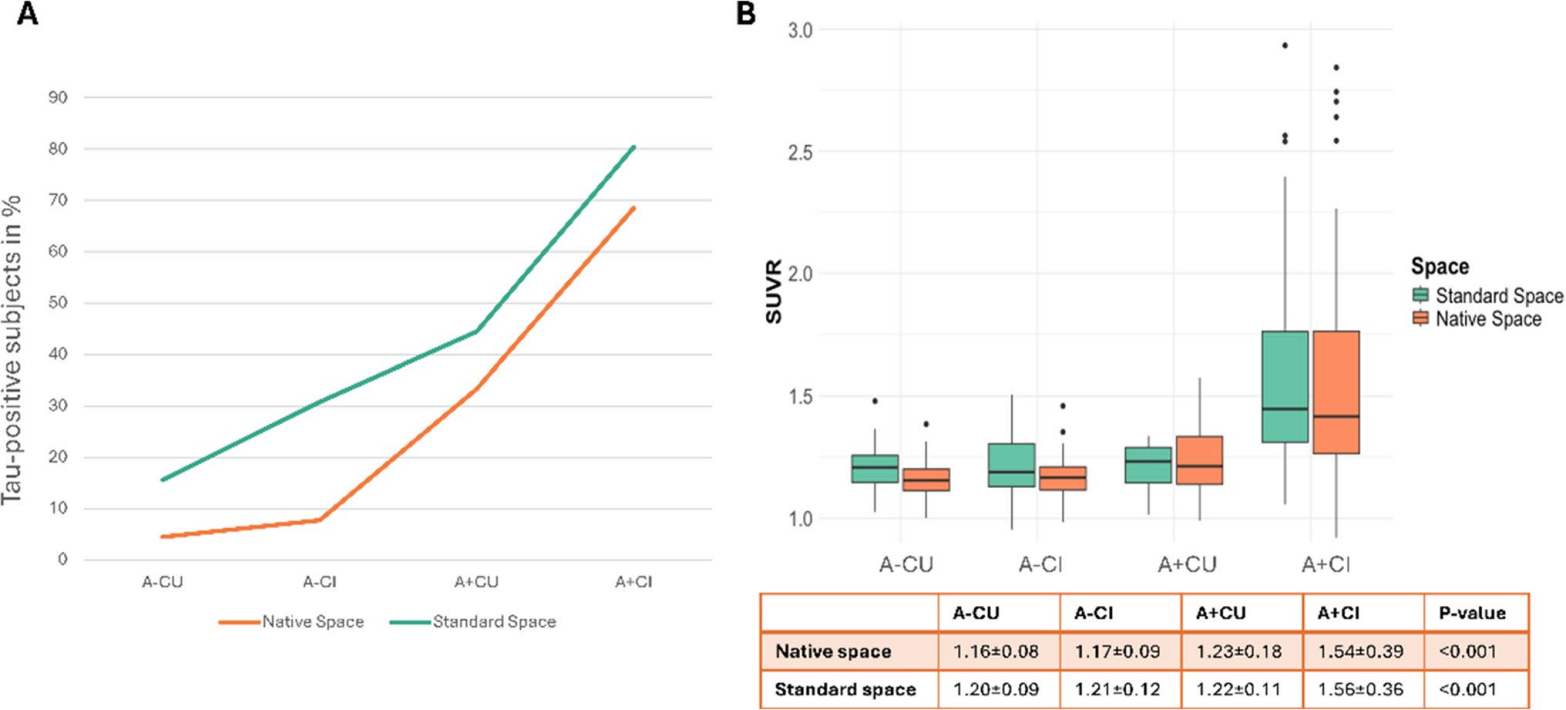
Tau status and quantification by different modalities. **A** Distribution of tau-positive patients in percentage for native and standard space methods in clinical stages. Tau positivity was defined according to the SUVR from early AD meta-ROI, threshold was set with Youden’s statistic. Number of subjects per group: A- CU: 45, A- CI: 39, A + CU:9, A + CI: 92. **B** Box plot of SUVR values from early AD meta-ROI region from native and standard space. Abbreviations: *A* amyloid, *CU* cognitively unimpaired, *CI* cognitively impaired, *SUVR* standardized uptake value ratio

When comparing the SUVR obtained from both methods, significant differences were found in all regions (*p* < 0.001), with the standard space approach yielding higher SUVR, with highest mean differences seen in Braak I and II (Table 2). Comparing the SUVR across clinical stages, there is a significantly increasing trend in SUVR for both methods along the AD spectrum, with standard space SUVR being systematically higher (Table 2). When we set threshold values for native and standard space, we found a SUVR cutoff of 1.3 for the native space and 1.28 for standard space method able to discriminate between tau-positive and negative cases. Across clinical stages, both native and standard space methods consistently detected a substantial increase in the rate of tau-positive cases along the AD continuum, and low tau positivity in non-AD (A-) (Fig. 2, panel A). When we examined tau positivity in non-AD individuals, we identified seven tau positive cases with the standard space approach in A-CU but all were visually classified as negative (two of them corresponding to Braak I). Among A-CI, 12 individuals were tau positive and 11 of them were visually classified as negative (2 of them were at Braak I) and one individual was visually classified as Braak V. In native space method, two tau positive cases were identified in A-CU group, both cases were classified as negative. Within A-CI group, we found two tau-positive cases and one was visually classified negative and the other as positive (Braak V). Overall standard space method identified more false positive cases compared to native space approach in all groups (Figure S3). Examples of discordant classifications (based on visual assessment and on the standard and native processing) are presented in Figure S4. Between clinical stages, SUVR for the two methods did not differ significantly (Fig. 2, panel B). However, the increase of SUVR values within the methods across clinical stages was significant (Fig. 2, panel B).

**Table 2:** Mean SUVR values obtained using native space and standard space methods

	Standard space	Native space	Mean difference	*P*-value
MTL	1.46 ± 0.3	1.38 ± 0.3	0.07	< 0.001
Early AD meta-ROI	1.39 ± 0.3	1.36 ± 0.3	0.02	0.003
Global AD meta-ROI	1.49 ± 0.4	1.45 ± 0.4	0.04	< 0.001
Lateral Temporal	1.42 ± 0.4	1.38 ± 0.4	0.04	< 0.001
Braak I	1.49 ± 0.4	1.38 ± 0.3	0.11	< 0.001
Braak II	1.45 ± 0.3	1.34 ± 0.2	0.11	< 0.001
Braak III & IV	1.42 ± 0.3	1.34 ± 0.3	0.07	< 0.001
Braak V & VI	1.25 ± 0.3	1.21 ± 0.3	0.04	< 0.001

*MTL* medial temporal lobe, *AD* Alzheimer’s Disease, *ROI* region of interest

When we assess the performance of SUVR obtained with the two processing methods in discriminating visually established tau status, both demonstrated high AUC values, across all regions. However, some significant differences were observed with the native space method showing stronger discriminative power over the standard space approach for all the regions (Table 3). Both methods showed high AUC results in discriminating A + CI from A-CU (AUC > 0.7), and no significant differences were found between native and standard space processing. When discriminating decliner and stable individuals, SUVR in the MTL obtained with native space processing method outperformed the one obtained with standard space method (*p* = 0.04) (Table 3).

**Table 3:** Diagnostic performance of native and standard space processing in all meta-ROI regions and Braak stages to identify visually assessed tau status, to distinguish amyloid-positive cognitively impaired from amyloid-negative cognitively unimpaired and to identify subjects declining over time

	Space	Tau Visual AUC	Z score & *p*-value	Amyloid & Diagnosis AUC	Z score & *p*-value	Declining AUC	Z score & *p*-value
MTL	Native Standard	0.915* 0.857	Z = -3.76 P = 0.0002	0.914 0.891	Z = -1.16 P = 0.25	0.810* 0.765	Z = -2.03 P = 0.04
Early AD meta-ROI	Native Standard	0.958* 0.935	Z = -2.18 P = 0.03	0.900 0.888	Z = -0.71 P = 0.48	0.816 0.810	Z = -0.29 P = 0.77
Global AD meta-ROI	Native Standard	0.957* 0.923	Z = -2.87 P = 0.004	0.911 0.896	Z = -0.80 P = 0.42	0.828 0.802	Z = -1.23 P = 0.22
Lateral Temporal	Native Standard	0.951* 0.909	Z = -3.12 P = 0.002	0.886 0.863	Z = -1.08 P = 0.28	0.816 0.794	Z = -0.87 P = 0.38
Braak I	Native Standard	0.929* 0.885	Z = -3.32 P = 0.0008	0.910 0.905	Z = -0.30 P = 0.76	0.811 0.770	Z = -1.86 P = 0.06
Braak II	Native Standard	0.799* 0.737	Z = -3.18 P = 0.001	0.831 0.802	Z = -1.33 P = 0.18	0.736 0.693	Z = -1.60 P = 0.11
Braak III & IV	Native Standard	0.944* 0.890	Z = -3.43 P < 0.001	0.898 0.866	Z = -1.35 P = 0.18	0.822 0.793	Z = -1.16 P = 0.24
Braak V & VI	Native Standard	0.882* 0.812	Z = -2.82 P = 0.005	0.811 0.755	Z = -1.57 P = 0.12	0.776 0.742	Z = -0.78 P = 0.43

*MTL* medial temporal lobe, *AD* Alzheimer’s Disease, *ROI* region of interest, *AUC* area under the receiver operating characteristic curve

Tau status based on visual read, Z scores and p-values are from the DeLong test, * refers to the significant values

Similar results were demonstrated when analyzing a subgroup of scans acquired with the Biograph mCT scanner (Table S1), showing significantly higher discriminative power of native space processing over standard space in classifying tau positive and tau negative cases. High discriminative power were found for both methods in distinguishing A + CI from A-CU, with no significant difference between the two methods. In discriminating decliners and non-decliners there was no significant difference between the two processing methods (Table S1). For scans acquired using the machine with higher sensitivity and resolution, namely Biograph Vision 600 Edge, there was no significant difference between native and standard processing methods in discriminating visual tau status, A + CI/A-CU status, and declining status (Table S2).

When we compared SUVR obtained with the standard space processing methods with two different reference regions, we found strong correlations between two approaches (Figure S1) and only marginal differences in discriminating patients in AD continuum (Table S3). In particular SUVR obtained using the cerebellar crus as reference region were able to better discriminate tau visual status only in MTL and Braak I. In contrast, inferior cerebellum grey matter was superior in discriminating A + CI from A-CU in early AD meta-ROI (Table S3). All these results are fully reported in the Supplementary Materials.

### Correlations with plasma biomarkers

**Fig. 3:**
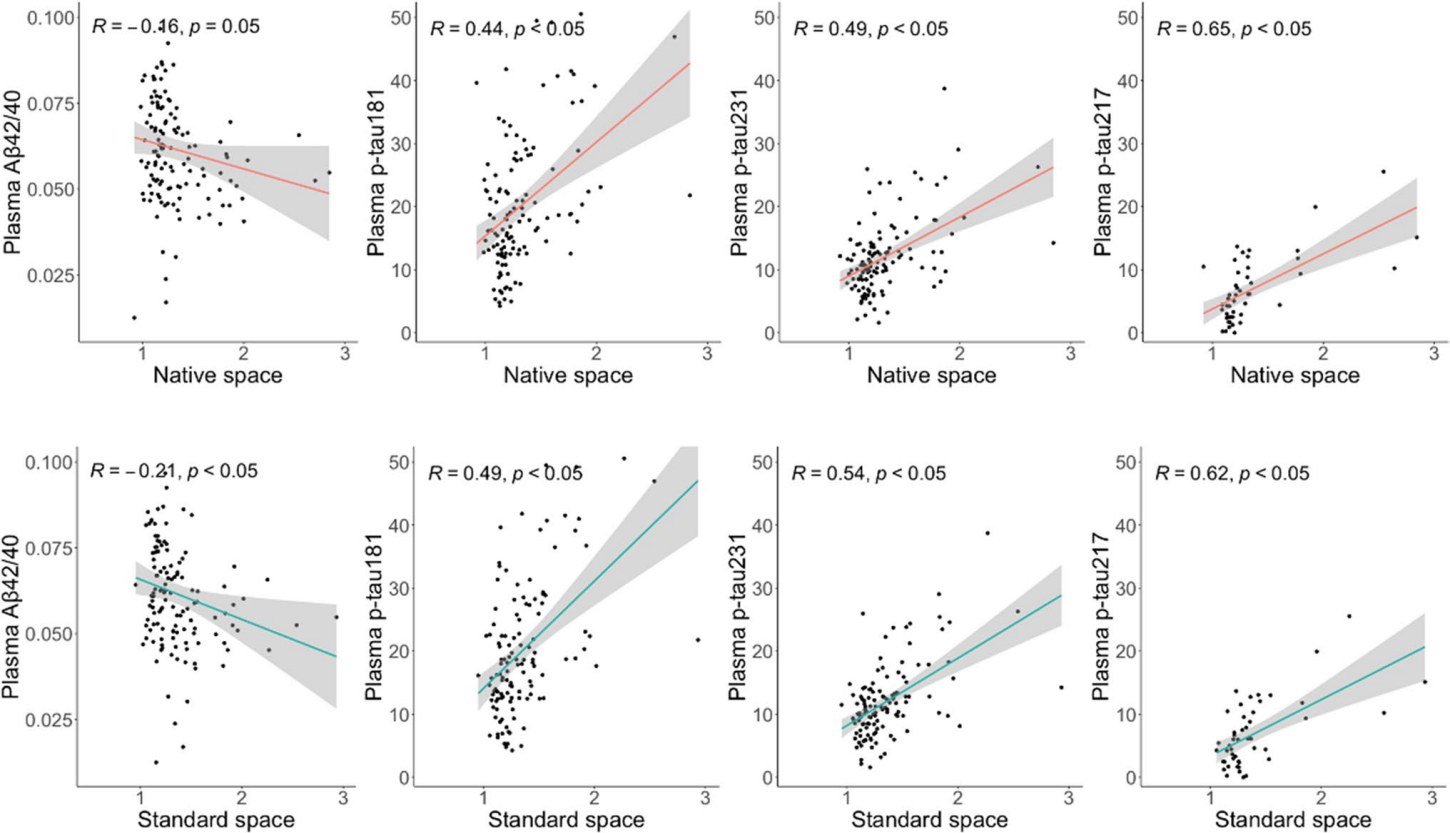
Correlations between plasma biomarkers and tau SUVR. Scatter plots between plasma biomarkers and SUVR values from early AD meta-ROI from native space and standard space methods. Abbreviations: *Aβ* Amyloid Beta, *AD* Alzheimer’s disease, *ROI* region of interest

Significant associations were found between all tau plasma biomarkers and early AD meta-ROI SUVR obtained with both methods (*p* < 0.05) in the whole population. P-tau217 presented higher correlation coefficients (r > 0.6) than other plasma biomarkers using both methods (Fig. 3). Correlations between plasma biomarkers and early AD meta-ROI SUVR were not different between the two processing methods (*p* > 0.05) [24]. Comparable correlations were found using SUVR from global AD meta-ROI instead of early AD meta-ROI.

When we tested the standard space method using the cerebellar crus as reference regions, we found comparable correlations results with plasma biomarkers (Figure S2).

## Discussion

The present study investigated the comparability of two distinct preprocessing methods for quantifying ^18^F-flortaucipir PET and for discriminating patient groups in AD continuum. We aimed to assess whether native and standard space methods produce similar results, as the standard space method represents a faster method applicable also when MRI data is absent. The two approaches demonstrated strong and significant correlations in all considered AD-related regions and a similar capacity in differentiating AD patients from non-AD patients. Although two pipelines could capture similar overall tau deposition patterns in AD, some differences emerged favouring the native preprocessing being similar to visual reading and more accurate in quantifying tau in the regions representing the earliest disease stages.

The SUVR obtained from both preprocessing methods showed strong correlations across all regions, indicating a high consistency in quantifying tau and supporting robustness of both methods for detecting overall tau pathology. Our findings indicate increase in tau-positive cases in both methods along the AD spectrum, with SUVR progressively increased from A-CU to A + CI individuals, reflecting a well-established link between tau accumulation and clinical symptoms, regardless of the reference space selected in the processing pipeline, further supporting the value of tau-PET as staging marker in AD [25]. In non-AD individuals, standard space method tends to report many false tau-positive according to visual standard, meanwhile the ratio between semi-quantification and visual reads was more consistent for native space approach.

Both approaches showed a good ability to discriminate AD patients, defined as A + CI, visually tau-positive or cognitively decliners, as tested by ROC analyses. Specifically, both methods demonstrated strong discriminative performance between tau status as assessed visually across all regions presenting high AUC (> 0.7); however, the native space method showed superior discriminative power with significantly greater values in all the regions. Previous research suggested high accuracy of tau visual assessment in identification of tau pathological change and validation of the results with postmortem information [26]. In MCI and AD, visually assessed tau positivity was associated with an increased risk of future cognitive decline [25]. Despite both preprocessing methods showing a very good performance in discriminating tau status, the native space preprocessing performed significantly better. In line with that, SUVR in MTL obtained with native space method performed better in discriminating cognitively declining participants from stable individuals compared to the standard space method. MTL is known to be the earliest site of tau accumulation making the native space method potentially more sensitive to subtle, early-stage tau pathology [2]. The native space approach may more accurately capture atrophy in small structures such as the entorhinal cortex. Using native space processing can improve the accuracy of regional quantification by preserving subject specific details and thus it can enhance sensitivity and reliability in both research and clinical settings. The highest added value resides in the identification of tau accumulation in the earliest stages. Analysing tau in the hippocampus and in MTL using ^18^F-flortaucipir PET poses a challenge due to off-target binding in the nearby choroid plexus. The choroid plexus signal occurs in the absence of tau and it likely has other causes such as iron deposition, melanin, and mineralization [27, 28]. In semi-quantification, adjusting for off-target binding can be difficult. Native space method may offer greater accuracy in discrimination of true binding and off-target signal since the ROI segmentation can be more precise compared to standard space. We found that the standard space method tends to overestimate SUVR likely due to the inclusion of off-target binding [10, 29]. Although the overperformance of native preprocessing on standard preprocessing was confirmed when limiting the analyses to the images acquired with a Biograph mCT scanner, no significant differences were found between native and standard space processing when images were acquired with the Biograph Vision 600 Edge scanner. The higher resolution and sensitivity of the Biograph Vision 600 Edge scanner enhances imaging accuracy and thus reduces the performance gap between the native and standard space processing, making both methods comparable even for quantification in small regions such as MTL.

Although the primary focus of our paper was on how different reference spaces can influence tau quantification, we are aware that the cerebellar crus is frequently used as a reference region for tau in standard space preprocessing [25]. Therefore, we examined this method in parallel to assess how it might differ between using cerebellar crus and inferior cerebellar grey matter as reference region in quantification of tau. Results showed no major differences between the methods. There was a strong correlation between the two approaches and overall, both methods showed similar clinical performance in discriminating AD patients, suggesting that both reference regions are valid and produce comparable and similar outcomes.

Given the emerging relevance of blood biomarkers, we tested if different methods showed similar correlations with these biomarkers as external measures of AD pathology. The correlations between plasma biomarkers and early AD meta-ROI SUVR show similar significant correlations using both methods. The biomarkers showed moderate to strong positive correlations indicating that higher plasma tau levels were associated with higher SUVR. We found the strongest correlations between p-tau217 and tau-PET, regardless of the preprocessing, and there were no differences in correlation coefficients between the two methods. In previous research, plasma p-tau217 was more effective than p-tau181 and p-tau231 in identifying tau positivity assessed by ^18^F-flortaucipir PET [30]. P-tau217 also showed stronger associations with global tau SUVR measured by tau-PET, and with global cognition assessed with the MMSE in a memory clinic population compared to other plasma biomarkers [31]. In head-to-head comparison of plasma biomarkers in prodromal AD, p-tau217 had higher accuracy compared to other p-tau biomarkers when identifying patients with abnormal Aβ and tau status and those who are progressing to AD [30, 32]. Our findings align with previous research, demonstrating significant associations with plasma biomarkers and early AD meta-ROI SUVR values computed using either the native space or standard space methods.

### Strengths and limitations

This study provides novel insights into the impact of processing space on ^18^F-flortaucipir PET quantification, a topic that has not been investigated before. One of the strengths of this study is the use of memory clinic population which ensures the translation of our findings into clinical practice.

However, some limitations should be acknowledged. Although we chose to compare the most used and well-known methods for tau quantification, we are aware that there are other reference regions, different techniques and different tracers that can be used for tau quantification. Moreover, we did not have brain tissue from postmortem evaluation to set as a gold standard.

## Conclusion

The present study shows that native space and standard space tau-PET processing methods perform comparably well in identifying AD patients. However, native space preprocessing performs more similar to visual reading and allows a more precise semi-quantification in the mesial temporal region, suggesting that it should be preferred in studies focusing on early tau deposition.

## Supplementary Information

Below is the link to the electronic supplementary material.

Supplementary file1 (DOCX 2.28 MB)

## Data Availability

Anonymized data used in this study are available upon reasonable request from the corresponding author (CB).
