# Supplementary material for: Comparison of two preprocessing methods for 18F-Flortaucipir PET quantification in Alzheimer’s disease
Source: Eur J Nucl Med Mol Imaging. 2025 Jul 19;53(1):480–90. doi: 10.1007/s00259-025-07452-3 (PMC12660454; doi:10.1007/s00259-025-07452-3)
Supplement: Supplementary file 1 — Supplementary file1 (DOCX 2.28 MB) [file 259_2025_7452_MOESM1_ESM.docx]

**Supplementary Materials**

**S1. MRI and PET acquisition**

T1-MRI images were performed at Division of Radiology of Geneva University Hospitals using a 3 Tesla scanner (Magnetom Skyra, Siemens Healthineers, Erlangen, Germany). T1-weighted Magnetization-Prepared Rapid Acquisition gradient Echo (MPRAGE) sequence was used. Acquisition parameters used were: TR, 1810 ms; TI, 900 ms; TE, 2.19 ms; flip angle, 8°; matrix size, 256x256; in-plane resolution of 1.0 x 1.0 mm2, slice thickness of 1.1 mm.

PET scans were acquired at the Division of Nuclear medicine and Molecular Imaging at Geneva University Hospitals using a Biograph 128 mCT (139 subjects), Biograph 128 Vision 600 Edge (66 subjects), or Biograph 64 TruePoint (4 subjects) PET scanners (Siemens Medical Solutions). For tau PET imaging, ^18^F-flortaucipir (FTP) was synthesized at the Centre for Radiopharmaceutical Sciences at ETH Zurich, Switzerland, under a license from the intellectual property owner (Avid, subsidiary of Lilly, Philadelphia, PA, USA). Participants received 197±39 MBq of FTP, and a late standard acquisition was performed 75 minutes post-injection, lasting 30 minutes. Each emission frame was reconstructed into six 5-minute frames and subsequently averaged into a single image. Tau distribution was visually assessed by nuclear medicine physicians, according to published recommendations [1].

Aβ-PET images were acquired using either (^18^F-florbetapir) FBP or (^18^F-flutametamol) FMM tracers. In the case of FBP, images were obtained 50 minutes after the intravenous administration of 210±18 MBq, consisting of 3x5 minute image frames. For FMM, images were acquired 90 minutes after the intravenous administration of 166±16 MBq, involving 4x5 minute image frames. Subsequently, the images were averaged to create a single frame over either 15 (FBP) or 20 (FMM) minutes. All Aβ-PET images were visually assessed by expert nuclear medicine physicians applying the standard operating procedures approved by the European Medicines Agency (<https://www.ema.europa.eu/en/documents/product-information/vizamyl-epar-product-information_en.pdf> ; <https://www.ema.europa.eu/en/documents/product-information/amyvid-epar-product-information_en.pdf>).

For all tracers, data were acquired in list mode and reconstructed using 3D OSEM (Ordered Subset Expectation Maximization). The reconstruction process involved corrections for randoms, dead time, normalization, scatter, attenuation, and sensitivity. After applying motion correction, a 2 mm Gaussian filter with a full width at half maximum (FWHM) was employed. The resulting images had a matrix size of 400x400 and isotropic voxels measuring 1.01 mm.

**S2. AD-related target regions**

After obtaining the SUVR for the Desikan-Killiany atlas regions with both processing methods, global tau SUVR were calculated from specific AD regions of interest (ROIs): (a) medial temporal lobe (MTL, including bilateral entorhinal cortex and amygdala), (b) early to later AD related areas (global AD meta-ROI) according to [2] (including bilateral entorhinal cortex, inferior temporal cortex, amygdala, middle temporal cortices and fusiform area), (c) early AD meta-ROI regions (including bilateral entorhinal cortex, lateral occipital cortex, inferior temporal cortex and amygdala) which are most discriminative for tau status for cognitively normal population according to [3], (d) lateral temporal ROI including superior temporal cortex, middle temporal cortex, and inferior temporal cortex, (e) and Braak regions (Braak I: bilateral entorhinal cortex; Braak II: hippocampus; Braak III-IV: parahippocampal gyrus, fusiform area, lingual area, amygdala, middle temporal cortices, caudal anterior cingulate, rostral anterior cingulate, posterior cingulate, isthmus cingulate, insula, inferior temporal gyrus, temporal pole; Braak V-VI: superior frontal, lateral orbitofrontal, medial orbitofrontal, frontal pole, caudal middle frontal, rostral middle frontal, rostral middle frontal, pars opercularis, pars obitalis, lateral occipital, supramarginal, inferior parietal, superior temporal, superior parietal, precuneus, bankssts, transverse temporal, pericalcarine, post central, cuneus, precentral, paracentral). SUVR in meta-ROIs and Braak regions were calculated using a weighted approach, mean among all included regions, accounting for the volume (native space processing) or the size (standard space processing) of each region.

**S3. Plasma biomarkers**

Plasma was collected in Ethylenediaminetetraacetic acid (EDTA) tubes and centrifuged (1700 g, 15 min). Samples were then aliquoted into 1.5 ml polypropylene tubes and stored at -80 °C in the Memory Clinic at Geneva University Hospitals. Aliquots were sent to University of Gothenburg for analysis. Plasma p-tau181 and p-tau231 concentrations were measured using in-house Single molecule array (Simoa) assays, as previously described [4,5]. Plasma Aβ42 and Aβ40 concentrations were measured using a commercially available Simoa assay (Quanterix, Billerica, MA). All Simoa assays were performed on an HD-X Analyzer (Quanterix, Billerica, MA) at the University of Gothenburg. Plasma p-tau217 concentration was measured at Lund University using a Meso Scale Discovery (MSD)-based immunoassay developed by Lilly Research Laboratories [6].

**Figure S1** Scatter plots between cerebellar crus and inferior cerebellar gray matter as reference region in processing across meta-ROI and Braak regions for the whole sample

Abbreviations: *MTL* medial temporal lobe, *AD* Alzheimer’s disease, *ROI* region of interest


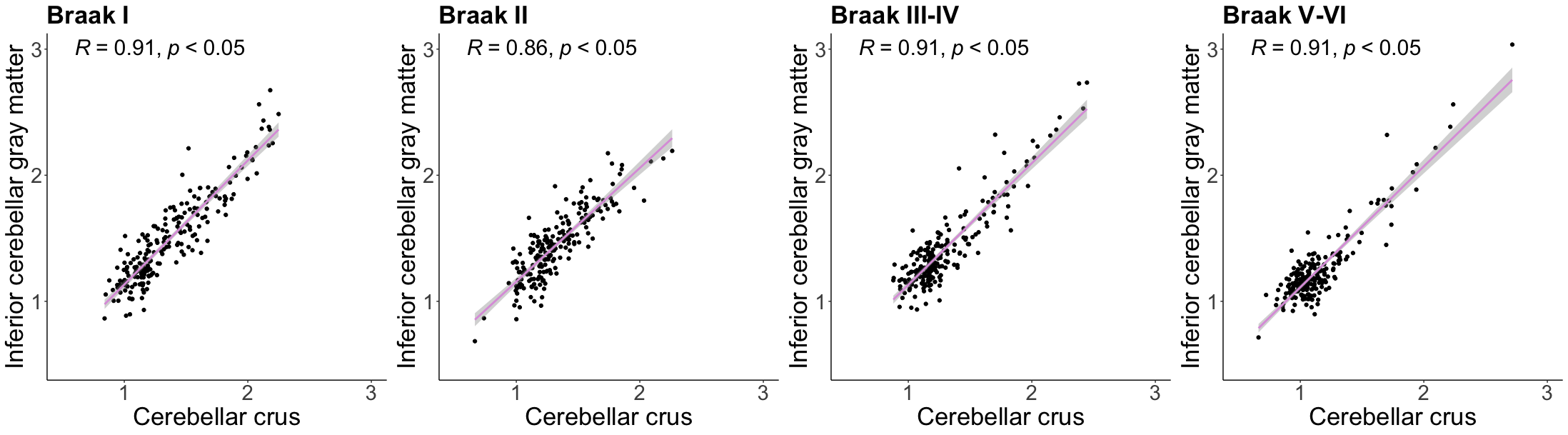

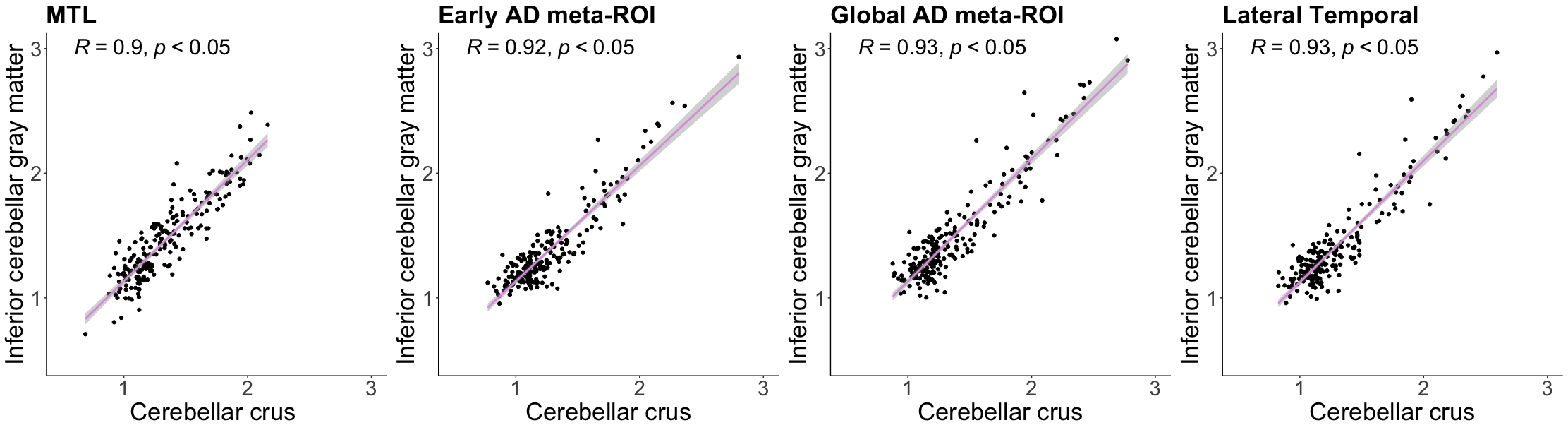


**Figure S2** Scatter plots between plasma biomarkers and SUVR values from early AD meta-ROI obtained using the (A) inferior cerebellar grey matter and (B) cerebellar crus as reference regions in the 2 preprocessing.

Abbreviations: *AD* Alzheimer’s disease, *ROI* region of interest, GM grey matter


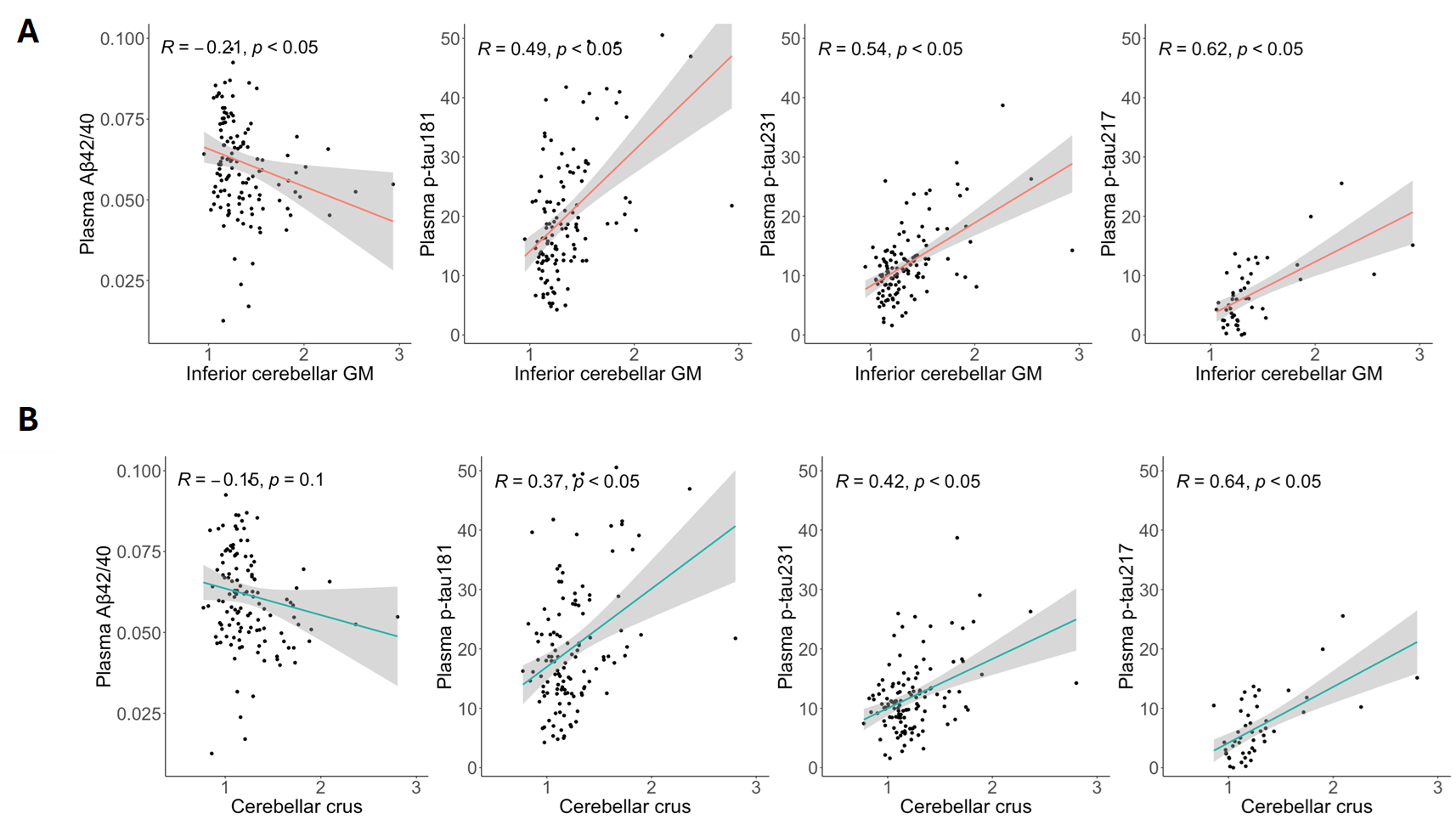


**Figure S3** False positive and false negative cases across visual tau assessment, standard space tau processing, and native space tau processing


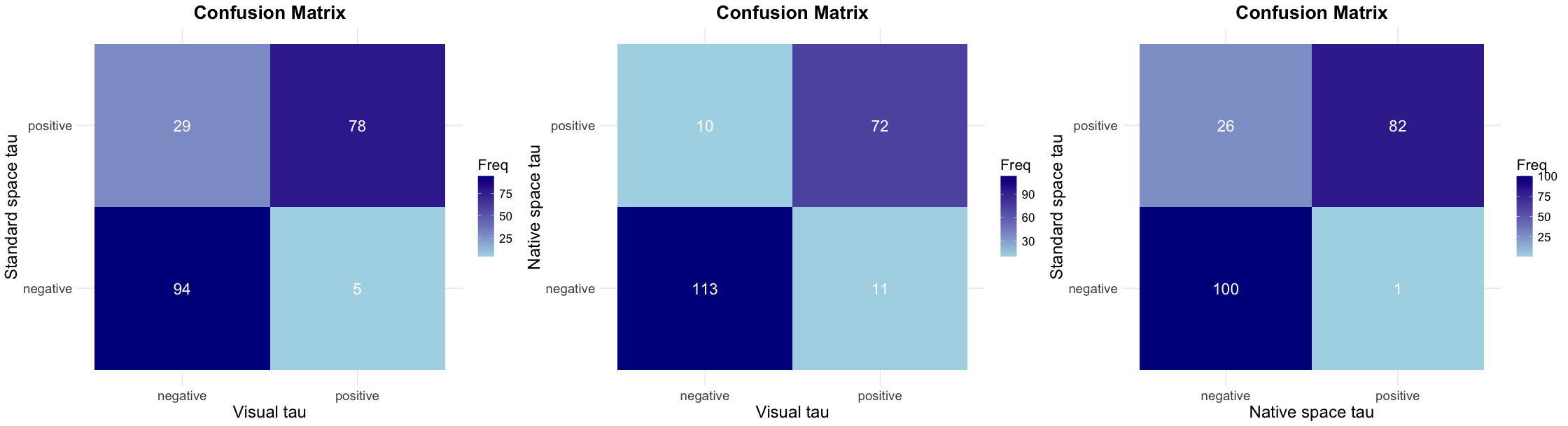


**Figure S4** Examples of each observed configuration for discrepant cases between tau status based on visual assessment and semi-quantification in native space and standard space processing


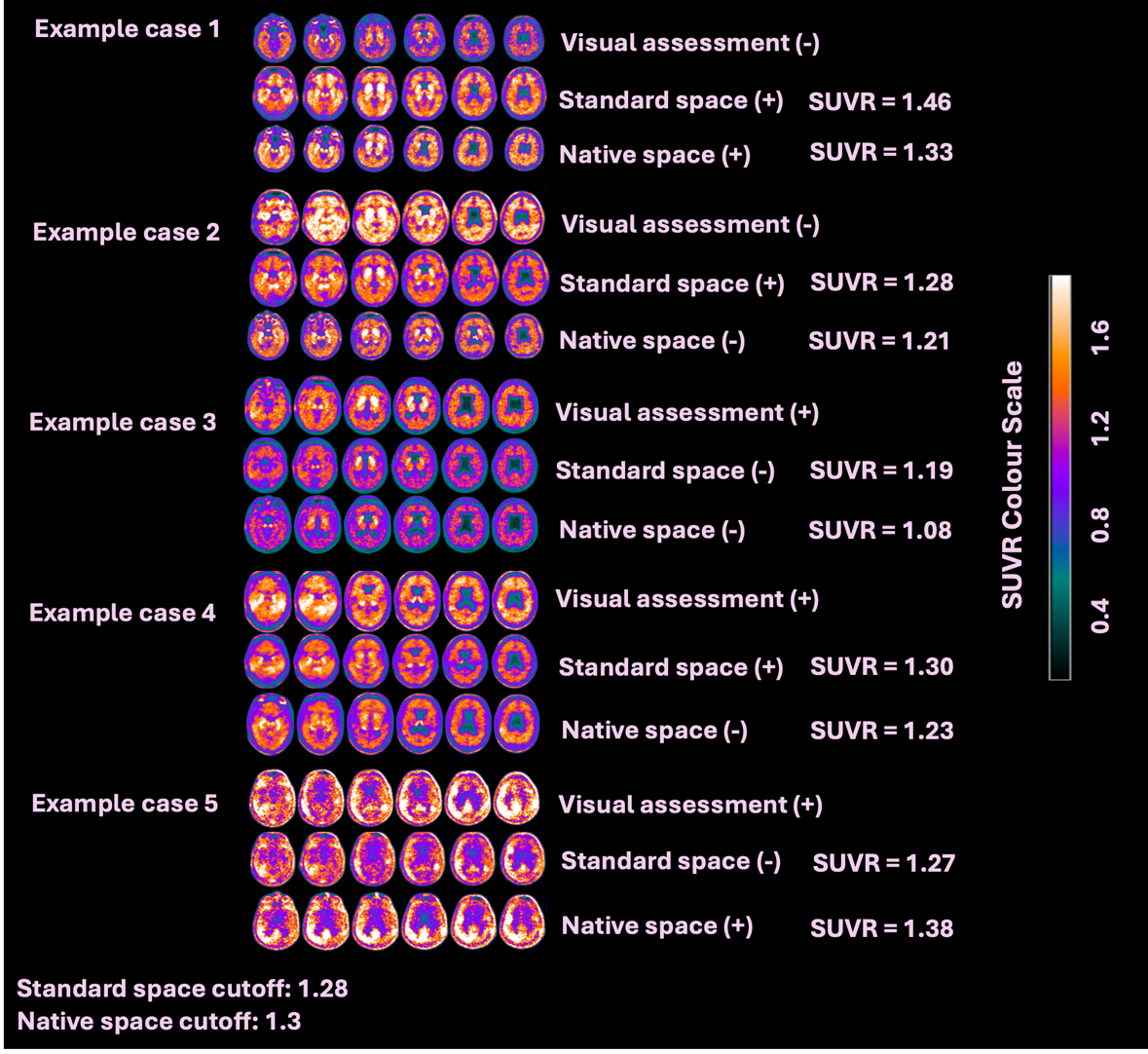
Abbreviations: *SUVR* standardized uptake value ratio

**Table S1** Diagnostic performance of native and standard space processing in all meta-ROI regions and Braak stages within a subgroup of subject scans acquired with Biograph mCT Siemens Medical Solutions (N= 66) to identify visually assessed tau status, to distinguish amyloid-positive cognitively impaired from amyloid-negative cognitively unimpaired and to identify subjects declining over time

|  | Reference region | Tau Visual AUC | Z score & p-value | Amyloid & Diagnosis AUC | Z score & p-value | Declining AUC | Z score & p-value |
| --- | --- | --- | --- | --- | --- | --- | --- |
| MTL | Native  Standard | 0.957  0.886* | Z = -2.58  P = 0.009 | 0.992  0.937 | Z = -1.85  P = 0.06 | 0.904  0.835 | Z = -1.72  P = 0.08 |
| Early AD meta-ROI | Native  Standard | 0.986  0.961 | Z = -1.47  P = 0.14 | 0.934  0.904 | Z = -0.91  P = 0.36 | 0.922  0.901 | Z = -0.68  P = 0.49 |
| Global AD meta-ROI | Native  Standard | 0.991  0.932* | Z = -2.19  P = 0.03 | 0.961  0.909 | Z = -1.50  P = 0.13 | 0.945  0.904 | Z = -1.44  P = 0.15 |
| Lateral Temporal | Native  Standard | 0.974  0.933* | Z = -2.06  P = 0.04 | 0.948  0.887 | Z = -1.57  P = 0.12 | 0.936  0.899 | Z = -1.43  P = 0.15 |
| Braak I | Native  Standard | 0.957  0.899* | Z = -2.12  P = 0.03 | 0.986  0.950 | Z = -1.47  P= 0.14 | 0.893  0.817 | Z = -1.99  P = 0.05 |
| Braak II | Native  Standard | 0.858  0.777* | Z = -2.47  P = 0.01 | 0.904  0.848 | Z = -2.07  P = 0.38 | 0.774  0.710 | Z = -1.24  P = 0.22 |
| Braak III & IV | Native  Standard | 0.986  0.912* | Z = -2.35  P = 0.02 | 0.956  0.890 | Z = -1.52  P = 0.13 | 0.928  0.890 | Z = -1.19  P = 0.23 |
| Braak V & VI | Native  Standard | 0.914  0.860 | Z = -1.49  P = 0.14 | 0.857  0.780 | Z = -1.28  P = 0.20 | 0.896  0.835 | Z = -1.20  P = 0.23 |
| Abbreviations: *MTL* medial temporal lobe, *early AD meta-ROI* early Alzheimer’s disease region of interests, *global AD meta-ROI* global Alzheimer’s disease region of interests *AUC* area under the receiver operating characteristic curve  Tau status based on visual read, Z scores and p-values are from the DeLong test, * refers to the significant values  Out of 66 subjects 39 have clinical follow up | | | | | | | |

**Table S2** Diagnostic performance of native and standard space processing in all meta-ROI regions and Braak stages within all subject scans acquired with Biograph Vision 600 Edge Siemens Medical Solutions (N=66) to identify visually assessed tau status, to distinguish amyloid-positive cognitively impaired from amyloid-negative cognitively unimpaired and to identify subjects declining over time

|  | Reference region | Tau Visual AUC | Z score & p-value | Amyloid & Diagnosis AUC | Z score & p-value | Declining AUC | Z score & p-value |
| --- | --- | --- | --- | --- | --- | --- | --- |
| MTL | Native  Standard | 0.879  0.835 | Z = -1.92  P = 0.06 | 0.939  0.944 | Z = 0.15  P = 0.88 | 0.820  0.770 | Z = -0.93  P = 0.35 |
| Early AD meta-ROI | Native  Standard | 0.943  0.925 | Z = -0.92  P = 0.36 | 0.926  0.942 | Z = 0.57  P = 0.57 | 0.829  0.779 | Z = -1.18  P = 0.24 |
| Global AD meta-ROI | Native  Standard | 0.923  0.910 | Z = -1.03  P = 0.30 | 0.931  0.939 | Z = 0.27  P = 0.78 | 0.834  0.811 | Z = -0.88  P = 0.37 |
| Lateral Temporal | Native  Standard | 0.922  0.884 | Z = -1.86  P = 0.06 | 0.918  0.913 | Z = -0.15  P = 0.88 | 0.829  0.770 | Z = -1.27  P = 0.20 |
| Braak I | Native  Standard | 0.906  0.870 | Z = -1.74  P = 0.08 | 0.910  0.950 | Z = 0.86  P= 0.39 | 0.825  0.779 | Z = -1.16  P = 0.25 |
| Braak II | Native  Standard | 0.790*  0.719 | Z = -1.97  P = 0.04 | 0.905  0.936 | Z = -1.37  P = 0.17 | 0.737  0.705 | Z = -0.60  P = 0.55 |
| Braak III & IV | Native  Standard | 0.898  0.888 | Z = -0.68  P = 0.50 | 0.921  0.907 | Z = -0.33  P = 0.74 | 0.825  0.811 | Z = -0.54  P = 0.58 |
| Braak V & VI | Native  Standard | 0.867  0.828 | Z = -1.27  P = 0.20 | 0.881  0.780 | Z = -1.45  P = 0.15 | 0.834  0.802 | Z = -1.11  P = 0.27 |
| Abbreviations: *MTL* medial temporal lobe, *early AD meta-ROI* early Alzheimer’s disease region of interests, *global AD meta-ROI* global Alzheimer’s disease region of interests *AUC* area under the receiver operating characteristic curve  Tau status based on visual read, Z scores and p-values are from the DeLong test, * refers to the significant values  Out of 66 subjects 39 have clinical follow up | | | | | | | |

**Table S3** Diagnostic performance of cerebellar crus and inferior cerebellar gray matter as a reference region in standard space in all meta-ROI regions and Braak stages to identify visually assessed tau status, to distinguish amyloid-positive cognitively impaired from amyloid-negative cognitively unimpaired and to identify subjects declining over time

|  | Reference region | Tau Visual AUC | Z score & p-value | Amyloid & Diagnosis AUC | Z score & p-value | Declining AUC | Z score & p-value |
| --- | --- | --- | --- | --- | --- | --- | --- |
| MTL | Inferior cerebellar GM  Cerebellar crus | 0.857  0.901* | Z = 2.79  P = 0.01 | 0.891  0.908 | Z = 0.87  P = 0.38 | 0.765  0.790 | Z = 1.02  P = 0.31 |
| Early AD meta-ROI | Inferior cerebellar GM  Cerebellar crus | 0.935  0.920 | Z = -0.92  P = 0.36 | 0.888*  0.830 | Z = -2.22  P = 0.03 | 0.810  0.804 | Z = -0.16  P = 0.87 |
| Global AD meta-ROI | Inferior cerebellar GM  Cerebellar crus | 0.923  0.938 | Z = 1.04  P = 0.29 | 0.896  0.872 | Z = -0.91  P = 0.36 | 0.802  0.814 | Z = 0.39  P = 0.71 |
| Lateral Temporal | Inferior cerebellar GM  Cerebellar crus | 0.909  0.911 | Z = 0.13  P = 0.90 | 0.863  0.818 | Z = -1.43  P = 0.15 | 0.794  0.814 | Z = 0.56  P = 0.68 |
| Braak I | Inferior cerebellar GM  Cerebellar crus | 0.885  0.919* | Z = 2.57  P = 0.01 | 0.905  0.913 | Z = 0.44  P= 0.66 | 0.770  0.797 | Z = 1.29  P = 0.27 |
| Braak II | Inferior cerebellar GM  Cerebellar crus | 0.737  0.773 | Z = 1.68  P = 0.09 | 0.802  0.804 | Z = 0.07  P = 0.94 | 0.693  0.707 | Z = 0.43  P = 0.66 |
| Braak III & IV | Inferior cerebellar GM  Cerebellar crus | 0.890  0.917 | Z = 1.37  P = 0.17 | 0.866  0.859 | Z = -0.22  P = 0.82 | 0.793  0.815 | Z = 0.65  P = 0.52 |
| Braak V & VI | Inferior cerebellar GM  Cerebellar crus | 0.812  0.847 | Z = 1.16  P = 0.24 | 0.755  0.736 | Z = -0.45  P = 0.65 | 0.742  0.767 | Z = 0.52  P = 0.60 |
| Abbreviations: *MTL* medial temporal lobe, *early AD meta-ROI* early Alzheimer’s disease region of interests, *global AD meta-ROI* global Alzheimer’s disease region of interests *AUC* area under the receiver operating characteristic curve  Tau status based on visual read, Z scores and p-values are from the DeLong test, * refers to the significant values | | | | | | | |

**References**

1. Fleisher AS, Pontecorvo MJ, Devous MD, Lu M, Arora AK, Truocchio SP, et al. Positron Emission Tomography Imaging with [18F]flortaucipir and Postmortem Assessment of Alzheimer Disease Neuropathologic Changes. JAMA Neurol. 2020;77:829–39.

2. Ossenkoppele R, Rabinovici GD, Smith R, Cho H, Scholl M, Strandberg O, et al. Discriminative accuracy of [18F]flortaucipir positron emission tomography for Alzheimer disease vs other neurodegenerative disorders. JAMA - Journal of the American Medical Association. 2018;320:1151–62.

3. Mishra S, Gordon BA, Ph D, Su Y, Ph D, Christensen J, et al. AV-1451 PET Imaging of Tau Pathology in Preclinical Alzheimer Disease: 2017;171–8.

4. Karikari TK, Pascoal TA, Ashton NJ, Janelidze S, Benedet AL, Rodriguez JL, et al. Blood phosphorylated tau 181 as a biomarker for Alzheimer’s disease: a diagnostic performance and prediction modelling study using data from four prospective cohorts. Lancet Neurol. 2020;19:422–33.

5. Ashton NJ, Pascoal TA, Karikari TK, Benedet AL, Lantero-Rodriguez J, Brinkmalm G, et al. Plasma p-tau231: a new biomarker for incipient Alzheimer’s disease pathology. Acta Neuropathol [Internet]. 2021;141:709–24. Available from: https://doi.org/10.1007/s00401-021-02275-6

6. Doré V, Doecke JD, Saad ZS, Triana-Baltzer G, Slemmon R, Krishnadas N, et al. Plasma p217+tau versus NAV4694 amyloid and MK6240 tau PET across the Alzheimer’s continuum. Alzheimer’s and Dementia: Diagnosis, Assessment and Disease Monitoring. 2022;14:1–11.
